# ‘You have to do what is best’: The lived reality of having a child who is repeatedly hospitalized because of acute lower respiratory infection

**DOI:** 10.1111/hex.13408

**Published:** 2021-12-20

**Authors:** Karen McBride‐Henry, Charissa Miller, Adrian Trenholm, Tara N. Officer

**Affiliations:** ^1^ School of Nursing, Midwifery and Health Practice, Wellington Faculty of Health Victoria University of Wellington Wellington New Zealand; ^2^ Kidz First Hospital, Middlemore Hospital Counties Manukau District Health Board Auckland New Zealand; ^3^ Paediatrics, Child and Youth Health, School of Medicine, Faculty of Medical and Health Sciences University of Auckland Auckland New Zealand; ^4^ Health Services Research Centre, Wellington Faculty of Health Victoria University of Wellington Wellington New Zealand

**Keywords:** acute illness, childhood hospital readmissions, equity, family, lower respiratory infections, parenting, vulnerable people groups

## Abstract

**Introduction:**

Hospitalization of children is traumatic for children and their families. Little is known about the impact of repeated acute admissions on families, or of these experiences in Indigenous populations and ethnic minorities. This study explores the societal and health experiences for families who have a child under two years of age, admitted to hospitals more than twice for lower respiratory infections.

**Methods:**

Underpinned by a reflective lifeworld research methodology, this article presents results from 14 in‐depth interviews in Aotearoa/New Zealand.

**Results:**

Families learn to identify illness early and then navigate hospital systems. These families struggle to create safe spaces for their children at home or in society. Wider social and economic support are central to family resilience, without which they struggle.

**Conclusion:**

This study reinforces the importance of bringing meaningful, culturally‐responsive care to the fore of treatment, particularly when managing vulnerable minorities. Formal referral and support processes are key to this responsiveness to lessen the burdens of acute admissions for families.

**Patient or Public Contribution:**

Families chose to be involved in this study to highlight the importance of the topic and their experiences with accessing health care. The cultural advisors to the project provided feedback on the analysis and its applicability for the participant community.

## 1 INTRODUCTION

Lower respiratory infections (LRIs) are a significant health burden for Aotearoa/New Zealand's paediatric population,^1^ resulting in acute hospital readmissions, particularly for children in the first 2 years of life;^1^, ^2^ Indigenous Māori children and Pacific children (an underserved minority) have high hospitalization rates.^2^ Limited research has been conducted in this area of high childhood morbidity and acute hospitalization. Yet, there is growing evidence linking LRIs in early childhood to recurrent respiratory conditions including chronic bronchitis and increasing rates of bronchiectasis, potentially leading to chronic lung disease in early and later life.^3^, ^4^ The impact of LRI can, therefore, be life‐long from a social and health perspective.

Populations most likely to carry the burden of acute readmissions to hospitals during the first years of a child's life are generally lower socioeconomic status groups and ethnic minorities. Despite this, the experiences of these populations have received limited focus; researchers have explored, however, the experiences of parents whose children suffer chronic illnesses requiring frequent hospitalization. Brown^5^ reported on the coping mechanisms of Māori and Pacific families managing the continual re‐entry of their children into the health system with life‐threatening conditions. Her work suggests persistent barriers exist impacting on family engagement with the health system; at its core, the research indicates that family are critical resources for coping with these engagements.

Overseas, work in this area suggests that caregiver distress contributes directly to child health outcomes.^6^ Kepreotes et al.^7^ in their meta‐synthesis of qualitative literature between 2000 and 2009 on parenting a child with chronic health conditions, propose that parents' experiences caring for chronically‐ill children are similar irrespective of the condition. They experience grief about the diagnosis and recognize the on‐going and life‐long impact of chronic illness. Parents feel the need to master emotions, persevere and be vigilant for potential deteriorating health (see for example, Smith et al.,^6^ Beeton et al.,^8^ Nelson et al.,^9^ Resch et al.,^10^ Breen et al.^11^ and Hudson et al.^12^), while internalizing or pushing aside emotions around their child's illness.^13^, ^14^

Researchers have examined the impact on families of acute and unexpected intensive care unit (ICU) admissions of critically unwell children (see for example, Colville et al.,^15^ Abuqamar et al.,^16^ and Curtis et al.^17^). In their earlier systematic review of these cases, Shudy et al.^18^ emphasize how, during admission, parents and siblings are shocked and fearful, feelings hospitals intensify. Families stress the need for clear explanations of their child's treatment and better communication by ICU staff.^18^ Shudy et al.^18^ highlighted that in the long‐term, critical admissions may permanently change family relationships, although of note, there is less literature exploring this concept, but it is supported in a more recent 2020 update to this systematic review, which suggests impacts begin within 24 hours of admission and last for years after discharge.^19^ Similarly, environmental factors arising from childhood hospitalizations, including housing issues and parental financial or employment stressors arising due to their child's hospitalizations also negatively impact families.^20^

Previous studies have focused on parental involvement and support during a child's hospitalization.^12^, ^17^, ^21^, ^22^ Burke et al.^23^ and Kepreotes et al.^7^ emphasize the need for parents to manage relationships with health professionals (HPs). This tenuous process involved parents reluctantly taking charge and directing care provision. Parents discussed the fragility of their control over the situation, their need to be constantly attentive to their child's welfare and adopt a new sense of reality, which encompassed re‐envisioning the future for their family.

Parents have highlighted that they want HPs to provide practical ways of caring for and supporting their chronically‐ill child.^6^, ^11^, ^24^, ^25^ Such measures include nurses sharing parents' emotional burdens and supporting family coping strategies.^9^, ^26^ Assisting families in this way is important as positive health outcomes for chronically‐ill children are associated with functional family relationships.^6^ Similarly, support to navigate the health system and function within society (financially and through access to appropriate educational pathways), and to manage the immediate needs of ill children are also necessary if families are to function effectively whilst caring for their child.^6^, ^11^, ^12^

There is a dearth of research addressing the systemic impact on families of repeat hospitalizations of children with *acute* illnesses. Importantly, limited research has explored this phenomenon in minority populations who are more likely to experience societal and health problems leading to hospitalization.^27^ This article lays out results from a qualitative study exploring the societal and health consequences for families who have a child under 2 years of age, admitted to hospital more than twice for acute respiratory illness. Understanding the lifeworlds of these families facilitates the meaningful tailoring of health care services and interventions.

## 2 REFLECTIVE LIFEWORLD RESEARCH METHODOLOGY

This study follows the tenets of reflective lifeworld research. This methodology, explained extensively by Dahlberg et al.,^28^ involves seeking meaning from experiences. Applying this methodology enables researchers to see phenomenon afresh and readers to consider the material from their own perspectives knowing that there are multiple traditions, values, and beliefs influencing these perspectives. Three principles inform this methodology: the hermeneutic circle, historicity, and openness.

The hermeneutic circle emerged from Schleiermacher's work on hermeneutics.^28^, ^29^, ^30^ He posited that when engaged in the act of interpreting, it is important to consider the whole and the parts of a text, being consistently attentive to the minute, and the broader context to develop interpretations.^28^, ^31^ Emergent interpretations are never complete; they change as cultures and traditions advance.^31^, ^32^ Consequently, individuals are responsible for reinterpreting and engaging in hermeneutic circles to create new interpretations of experiences.

An individual's place in history affects their thinking, their history journeys with them and builds preunderstandings and interpretations about their environment. Individuals cannot understand or examine the future without first considering their traditions and previously held values; all future interpretations will emerge from this perspective or horizon.^31^, ^32^, ^33^ While it is impossible to free oneself from previously held ideas completely, researchers constantly question their historicity and vigilantly assess how these issues affect emerging interpretations.^31^ Through this position of ‘openness’, the researcher may then recognize the ‘otherness’ of a phenomenon.^31^, ^32^ Participants' lifeworlds are unique; understanding lived realities can inform meaningful dialogue around health care services and intervention, and therefore, is an appropriate methodology to underpin this study.

## 3 MATERIALS AND METHODS

**Figure 1:**
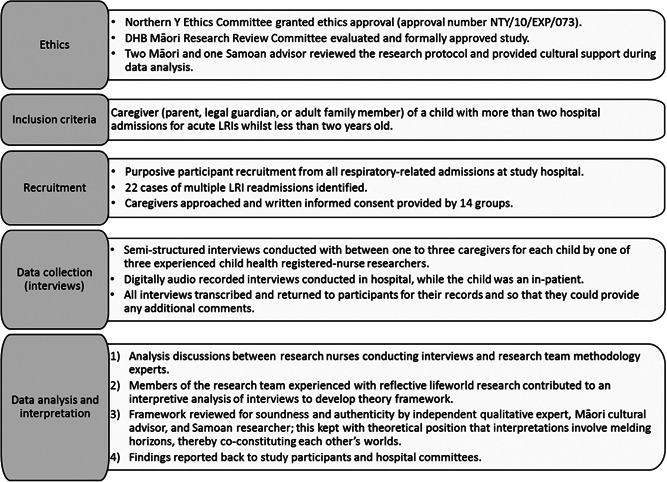
Data collection and analysis

This study (ethics approval number: NTY/10/EXP/073) was conducted in a children's hospital in the District Health Board region of South Auckland, Aotearoa/New Zealand. The 82‐bed hospital offers emergency and tertiary care for children under 14 years. Approximately 53% of this population is Māori or Pacific and 58% of the birth cohort live in the most socioeconomic deprived quintile of Aotearoa/New Zealand.^34^, ^35^ LRI, often bronchiolitis or pneumonia, is one of the most common causes of hospital admission for (Māori and Pacific) children in this region.^2^, ^36^ Issues contributing to high LRI rates include overcrowded and under‐heated housing.^36^ Figure 1 outlines key aspects of the research protocol, including for data collection, recruitment, ethics, and analysis, developed based on a recognition of South Auckland's unique population.

All 14 participant groups self‐nominated their ethnicity; most indicated they were of mixed ancestry. For example, participants stated they were of Māori and Niuean, Samoan and, or, Tongan, or Māori and European descent. Most participants resided in homes with their children and more than one adult and/or multigenerational households. Participants had two to six other children living in the family home (Table 1).

**Table 1:** Participant group demographics

S. no.	Ethnicity	Age	Relationship to child	Siblings hospitalized (frequency)	Adults at home (*n*)	Children at home (less than 5 years old)
1	Māori	20–25	Caregiver	No	8	3 (1)
2	Māori European	26–30	Mother and father	No	3	2 (1)
3	Samoan	31–35	Mother	No	4	2 (2)
4	Māori European Tongan	36–40	Father	No	5	2 (1)
5	Samoan Tongan	26–30	Mother and father	No	2	4 (2)
6	Samoan	26–30	Mother	Yes (1)	3	3 (2)
7	Samoan Tongan	31–35	Mother	Yes (10+)	3	4 (2)
8	Māori Niuean	20–25	Mother	Yes	5	4 (2)
9	Samoan	20–25	Mother	Yes	1	3 (3)
10	Māori	20–25	Mother	No	2	3 (3)
11	Tongan	41+	Mother	No	2	6 (1)
12	Māori Tongan	20–25 (Mother)	Mother and grandmother	No	5	5 (4)
13	Samoan	31–35	Mother	Yes (3)	7	8 (3)
14	Samoan	20–25	Mother	No	4	4 (2)

Participant recruitment and data collection were carried out by female research nurses (one Samoan and two of European descent), all had many years' experience working alongside South Auckland's population. To ensure consistency, before initiating data collection, the lead investigator provided training in qualitative interviewing. Interviews were conducted in English in several ways; for example, an interview may have been conducted with one caregiver, two parents together or a parent and grandparent. One interview involved three family members, both the child's parents and a grandmother. All interviews were conducted while the child was in hospital. Interview recordings were transcribed by a third party and then reviewed by the primary investigator.

Discussions between the three research nurses and two research team members occurred at the start of data analysis; these analysis discussions centred on the nurses' experiences with recruiting and interviewing participants and potential interpretive themes formed during their informal analysis. The transcripts were managed using NVivo11 (QSR International Pty Ltd). A formal interpretive analysis of interviews focused on illuminating meanings behind participant experiences. From this, an analysis framework was developed and reviewed for soundness and authenticity by the research team and cultural consultants to ensure a melding of horizons consistent with reflective lifeworld research methodology.

## 4 RESULTS

Three major themes emerged from the analysis, these are ‘coming‐to‐know’, ‘being in hospital’ and ‘navigating society’ (Table 2). Participants spoke of how, over time, they came to recognize and understand respiratory illness in their child, the factors causing illness, and how to protect their children from infection. They described coming to understand the hospital system but recognized they were considered outsiders, despite having expertize in caring for their child. Participants spoke at length on how they looked to improve housing situations to support their child's health. Across these themes, participants described the critical role extended families play in surviving readmissions.

**Table 2:** The thematic framework

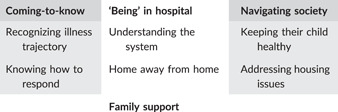

### 4.1 Coming‐to‐know

When participants spoke of their child's initial illness experience, they were often unable to recognize an illness trajectory. Gradually, participants came to know what respiratory illness was and the signs their child displayed. They were able to gauge illness severity and determine triggers in their child. The following narrative describes how one mother learnt to keep her child safe, and how she recognizes her son's repeat illnesses.

I pretty much say ‘no please like can you keep your guys' kids on that side don't bring him over here’ ‘cause I am scared [my son] might catch something, and then we have to rush back here [hospital]… I am very cautious everyone at home is very cautious of him. It is our main problem’ cause every time we see his chest come out then we all start freaking out. (Int.12)

The fear participants experienced when their children became sick was a compelling motivator to actively support their child's health. Parents often chose to keep their child socially isolated because of risks mixing with others presented; participants progressively gained this knowledge as they considered events preceding their child's latest LRI.

#### 4.1.1 Recognizing illness trajectory

In addition to understanding how respiratory illness presented in their child, participants learnt how the illness progressed. They saw what their child looked like when healthy and recognized downward acute LRI spirals, resulting in participants intervening before illness led to hospital admission.

When he is normal, he is fine, but I know when he is start [getting] sick, when he starts his breathing and his wheezing and I know that he has got [sick], and when he starts wheezing and I must be bringing him here [to hospital]. (Int.11)

This recognition of illness trajectory meant participants learnt how to respond when their child became ill. Despite this knowledge, sickness often led to emergency department visits. Once in the hospital, participants increasingly gained knowledge of the treatment their child needed.

#### 4.1.2 Knowing how to respond

Understanding that hospitalization was the ‘only option’ was a common narrative arising during interviews. One mother poignantly stated there was ‘no communicating with illness’, and that her child's condition ‘announced’ when it was time to return to the hospital.

I look at him and see he is not really okay… the only option for him is bringing him into the hospital… You don't communicate with a sickness that is coming, you don't know when it is coming, when it comes it comes so you have to prepare yourself for the outcomes and what is going to happen next… you have to do what is best [child coughing in background]. (Int.6)

This ability to recognize their children's response to illness was crucial to managing the illness and avoiding another acute admission. Participants consequently began to seek treatment sooner. Although unable to ‘communicate with an illness’, this distressing learning process provided participants with knowledge of how to respond to ill health.

### 4.2 ‘Being’ in hospital

Despite the challenges of having a frequently hospitalized child, the familiarity participants gained through repeated hospital exposure meant they understood what was happening and how to navigate ‘being’ in hospital. In some cases, it became a home away from home and a respite from direct responsibility for their child's illness. Participants were able to identify illness trajectories and seek timely treatment; however, their children did not always receive prompt care. Waiting for HPs to respond was often galling as participants had no control over hospital environments or service delivery. Treatment delays came at the expense of their child's health.

Mother: When we got there [to the hospital] her oxygen levels were at 92… and then half an hour, an hour later she was tested at like 89… in that time she had not been given any oxygen, she had not been given any inhalers… We could see her health declining…

Father: You have got to like [wish] their observational skills were quicker to assess what was wrong… you come into a place [a hospital] where you are actually supposed to look after people, and they cannot pick up on the signals [of illness in my child] when they are actually there to do it… If you don't want to do it, give me the tools to be able to do it myself, but they would not let me do that… Why did we come here [to the hospital]… [We] could have gotten more care at KFC [fast food outlet]. (Int.2)

Having faced repeated acute hospitalizations, participants understood where treatment was needed and how it should be managed; not receiving it resulted in deep frustration. They recognized delays as possibly prolonging their child's suffering and hospitalization. This came with other stressors, such as added time off work and childcare issues for other children. In addition to knowledge about LRIs, participants also learned how ‘to be’ in the hospital.

#### 4.2.1 Understanding the system

The frequency of hospitalization changed how participants perceived the hospital environment. At first, the environment was unfamiliar. Gradually, they appreciated the system, technology, and treatments, and they became more at ease in the space and able to adopt the role of parent–nurse.

[When she was first in hospital] seeing the other nurses tend to her and everything, and I just stood back and did not really take any part in it. I just let them do their job, but I learnt from everything that I had observed and kind of took that role upon myself, so I felt like a nurse and a mother… Knowing what the procedures are, I knew straight away what to do, where things are and what to use… Instead of waiting for the nurses I just go and do it myself instead of keep depending on them. (Int.10)

Understanding the hospital system empowered participants within an unfamiliar setting. This guided them to take control of caring for their children's complex acute needs. Through claiming an active contributor role, participants began to see the hospital as a second home.

#### 4.2.2 Home away from home

Given the many hospital admissions children in this study had, some parents found comfort in the hospital, describing it as like being at home. This stemmed partially from familiarity with hospital staff, but also because hospitalization meant respite for participants from direct responsibility for a child they had to fight to keep well.

Mother: This is like a second mortgage for us, like we live here, cause everyone knows him here, it is like we are just coming home.

Grandmother: We are coming home for a holiday.

4.2.3

Mother: …They have really good service, the people around here like staff are really good, they know I like it here… (Int.6)

Participants spoke about how they slowly acclimatized to hospital life, in the example above the mother referred to the hospital as a ‘second mortgage’, which may reflect, in part, the price of having an acutely unwell child. Participants highlighted that they gradually learnt to accept their child's hospitalization as necessary; they framed the hospital as a home away from home. This perspective on admissions allowed families to recast experiences as tools in caring for their child. That said, participants explained the need to navigate society to keep their child well and avoid future hospitalizations.

### 4.3 Navigating society

Participants spoke of how they worked hard to keep their children healthy and safe in society and away from the hospital. They used painful learnings gained from analysing the antecedents of their child's illness and learned to navigate their way around risks to keep their child well. Parents, therefore, kept their children away from others who might cause harm and attempted to address poor‐quality housing, a problem for almost all in our study.

#### 4.3.1 Keeping their child healthy

Participants learned that to keep their children well, they needed to guard their environments closely. This often meant that they limited their child's socialisation with other children to minimize the risk of infirmity. Participants found alternative arrangements for preschool, significantly affecting their children's social interactions. In the following account, a mother describes the role of Family Start,^37^ a programme where families with young children are visited in their homes and supported to learn about growth and development and keeping their child healthy.

With just five kids in the preschool, one child coughed and it went airborne and he caught it and we had to rush him back [to hospital]… We tried it again—same thing… Family Start… found a Māori correspondence school… but it took about four to five months to get him onto the course… cause they wanted to actually know… how he gets sick and why is it keeping him from going to preschool, primary school… we still kept all his admissions… and gave it to them and they said ‘this is the best for him’. (Int.12)

Sick children caused many changes in how their families interacted with society. Accessing and attending early childhood centres, an important social and developmental phase for most children was hazardous for these children. Outings, including going to grocery stores during winter, were viewed as threats to a child's health. To keep their child healthy, participants worked to address issues related to poor‐quality housing.

#### 4.3.2 Addressing housing issues

Participants discussed at length how poor‐quality housing affected their child's health and how home environments were fundamental to keeping their children healthy. They worked to ensure homes were warm and free of airborne contaminants through remedies, such as opening windows when cooking, and addressing complex family‐related factors. One example of managing the latter was the eviction of a partner, who smoked, from the home. One family spoke on the changes they engaged in to make their home function for their child; they offered the following narrative.

Mother: We have even changed the way we live at home, like the places we sleep. Me and [my son], we stay out in the back… We have had to even do changes to our house, we had to get the whole house insulated from the roof to the bottom, to getting a heat pump in, to humidify into the house, just to suck out all the bad air so he doesn't breathe it in. We have even had to stop the way we actually cook… Cause we know it can trigger it…

Grandmother: We build a new deck just for him, a deck for him to come out and play on it and a spare room for him, we have done all that… whatever he needs we try and get… (Int.12)

Changes made to the home environment were significant for families. Many lived in multigenerational groups with more than two adults; these families supported each other financially to update their homes. Even so, participants acknowledged the struggle to fund heating or repairs needed to keep their child well. Several were in rental accommodation, which meant they had little control over purchasing insulation or managing renovations.

### 4.4 Family support

Extended family was the primary support for participants when managing the challenges of a repeatedly acutely unwell child. Participants spoke of how their families took over caring for their other children, providing, amongst other things, food, clothing and housing. Strong family support became a way for households to survive with their wellbeing intact.

They [family] do all that for me, if I need new clothes or he needs clothes or something… We have got my nan, my aunty and my cousin in the main house, and then my uncle in the garage, and then us in the back house [I have lots of help at home]. (Int.1)

In addition to enabling care and support at home, families stepped in to care for other children when the primary caregivers were required in the hospital. In these situations, families managed responsibilities, sometimes for significant periods.

My mum was really supportive with me, she looked after my older daughter, she goes to school and I was here [in hospital] for three weeks… my uncle took my daughter to school while my mum was at work, and my sister was at school, and after work, they come along for a visit and bring along some food. (Int.6)

Family support enabled participants to create wellbeing, despite the challenging context that these households found themselves. Learning to use the hospital systems and manage their home to avoid future hospitalizations also presented challenges; however, family support enabled participants to manage their unwell child, other children and societal responsibilities.

One parent, a mother of three preschool children, had limited extended family support; she suffered significantly because of her child's repeat LRI admissions and was at the mercy of policies forcing her to place her young child alone in an ambulance, while trying to find care for her other children and when she was in the hospital caring for her unwell child. Financially, she struggled to feed her children and provide adequate housing. She also faced obstacles in managing relationships with others because of the situation with her child. Speaking at length about her experience, she described herself as being a bad mother and to blame for her child's sickness. She feared punishment and removal of her children by the State due to an insidious cycle of acute illness; this was a message reinforced by others she knew. She offered the following narrative.

I have to bring my family, cause once I have to put my son in an ambulance without me… and then I have to find who is going to come and look after my kids… I am a bad mum… It is really hard for me, and it is not my kids' fault, it is my fault for bringing them to life… I try to let all the social workers and doctors know what my situation is ‘cause I can't lose [custody of] one… It is hard for me, but I don't know what else I can do… I can't lose a baby’. (Int.9)

This narrative, and those highlighted earlier in this section, emphasize the role family support plays in helping participants manage their reoccurring acute LRI journey. Where family support exists, caregiver ability to respond to illness, ‘be’ in hospital, and navigate society are all positively affected. When this support is lacking, the burden of repeated sicknesses can be overwhelming.

## 5 DISCUSSION

Caring for a child frequently hospitalized over the first 2 years of their life is a challenging experience for participants. When these children first suffer from LRI hospitalizations, caregivers experience helplessness; over time, they learn skills and gain the experience needed when attending to their child whilst unwell. Participants discussed how they learnt to recognize the onset and trajectory of LRI; they became the unexpected expert. This meant they were able to seek treatment earlier and had insight into what hospitalization meant for them and their family. The process of gradually gaining illness knowledge has been explored within the context of chronic conditions^7^, ^23^ where researchers highlighted that parents slowly gain the knowledge needed to manage their child's illness. This study has revealed that the process of coming‐to‐know also exists for families with young children suffering from reoccurring acute LRIs.

The experiential knowledge participants gain, however, does not protect them from the childcare, housing, financial and employment pressures resulting from caring for an often‐unwell child. These are significant pressures for any family to address, particularly while remaining constantly vigilant for the next illness episode. Participants in the present study relayed similar narratives to parents of chronically unwell children (see for example, Kepreotes et al.^7^ and Burke et al.^23^). In Aotearoa/New Zealand, unlike families with chronically‐ill children, families of acutely unwell children cannot access state‐provided financial support. For example, they do not have access to disability allowances provided to support children with chronic illnesses. The hospital environment participants come to know so well, excludes their other children from staying on wards. This presents another significant burden as these parents may lack social capital or support, to find care for their other children. Solo parents are further penalized as this may mean having to leave a young child in the hospital alone. This issue has not emerged in earlier research examining acute childhood illness, although researchers have explored financial costs associated with acute care admissions.^20^ This study contributes insights into the holistic impact of acute hospitalization on the entire family.

Participants described hospitals as foreign environments that caused frustration. In time, they came to view the hospital as a home and likened it to having a ‘second mortgage’ because of their sense of familiarity and the respite they received from keeping their child safe. The narratives highlight the needs of this participant group and the duality between the hostile nature, as described in other research,^5^, ^19^, ^27^ and supportive nature of a hospital, creating an unresolvable conflict. Participants developed necessary skills to care for their child in a hospital setting earning the role of ‘parent‐nurse’. Burke et al.^23^ discuss how parents of chronically‐ill children ‘reluctantly take‐charge’ in moments where HPs fail to provide appropriate care. This current study confirms that parents with children suffering repeated acute LRIs engage in similar behaviour. The emerging narratives highlight how families are capable of proactive and knowledgeable care and are, in some cases, more responsive than HPs. HPs should recognize parental ability and intuition with their child and actively involve them as integral parts of the care team.

One potential solution for these children, who suffer repeated hospitalization with LRI, is for HPs to create action plans, like those used to manage patients with chronic asthma. This would enable the development of treatment protocols for when conditions worsen and would allow the management of social determinants influencing the health of children. Such plans have proven successful in reducing exacerbations in asthma patients.^38^, ^39^ This mechanism would empower parents to advocate for timely and appropriate treatment within primary and tertiary settings and would assist to reduce parental frustration with health care delivery. Creating and routinely updating such plans would help parents/caregivers and HPs navigate each other's role in the delivery of care and recognize the evolving nature of these roles. These plans should include a section prompting HPs to refer families to appropriate financial, educational, and social support services to address the wider issues contributing to repeated childhood LRI. Given the worldwide prevalence of COVID‐19 and the more detrimental effect on those with respiratory issues, formal planning and documentation of management plans for vulnerable children is particularly timely.

Participants were creative in addressing issues such as education and complex living arrangements as a way of managing LRI risk. To keep their children healthy, caregivers in the present study purposefully isolated themselves from wider society, as mixing with others brought risk of further infirmity. Chronic illness research has revealed that while parents perceived that the physical care of their children was met, there remained poor coordination of care and psychological support, affecting child and family wellbeing.^40^, ^41^ Consideration of the ongoing needs of children for such support is important. Failure to address these issues may further disadvantage children when entering formal education systems, particularly for children suffering from repeat hospitalizations at young ages.

Multigenerational homes are sometimes viewed negatively.^42^, ^43^ Participants in this study, however, indicated that these types of homes offered invaluable support and strength. Extended family members were able to care for the sick child's siblings and these children did not have their daily lives or routines disrupted. Housing quality and overcrowding are significant issues in Aotearoa/New Zealand, with Māori and Pacific children suffering more because of a lack of suitable housing.^44^ Public health initiatives to improve housing conditions will no doubt assist children living in substandard homes to stay well. However, initiatives aimed at reducing overcrowding, also need to encompass culturally‐appropriate dwellings, so that extended families can reside in the same home if they choose; a call also made by others.^45^, ^46^

Where family support exists, issues with responding to childhood illness, managing the hospital system, and navigating society appear to be mitigated. Similar results within the context of chronic illness and health care access have been discussed;^47^, ^48^ however, the challenges for single unit families, such as those that emerged in this study have not previously been explored. This study presents new knowledge, not only for managing acute care, but also for Indigenous and minority ethnic groups where extended families can offer social protection. It is acknowledged that this is a qualitative study that has inherent limitations because of its small sample size, single hospital site, and unique participant population group. Future research should consider the impact family structure has on a child's health care journey when managing acute conditions. Research in this area, and testing of the previously suggested action plans, may inform decisions on how best to use the unexpected expert.

## 6 CONCLUSION

This study gives voice to an under‐researched group of families and their children who are less than 2 years old and suffer from repeated acute LRI hospitalizations. Interpretation of participant narratives reflects the authors' reflective lifeworld journeys. Readers of this interpretation should engage in hermeneutic circles to create their own interpretations.

Participants described how over time they learned to recognize their child's LRI illness trajectory, becoming ‘parent‐nurses’ as they navigated the hospital system. Due to the burden of this illness, parents isolated their children from society, protecting them from health risks. They relied on multigenerational housing and extended family networks to survive but struggled with emotional, financial and familial burdens. The researchers recommend adopting action plans, traditionally used for asthma management, to empower families during acute admissions while enabling HPs to refer to appropriate support agencies. At its core, this article suggests the need for HP accountability in working with families to control ongoing‐acute conditions.

## CONFLICT OF INTERESTS

The authors declare that there are no conflict of interests.

## ETHICS STATEMENT

Ethics approval was granted by Northern Y Ethics Committee (approval number NYT/10/EXP/073). Written consent was obtained from all participants.

## AUTHOR CONTRIBUTIONS

*Conception and design*: Karen McBride‐Henry and Adrian Trenholm. *Acquisition of data*: Charissa Miller and Karen McBride‐Henry. *Methodology*: Karen McBride‐Henry. *Formal analysis*: Karen McBride‐Henry, Charissa Miller and Tara N. Officer. *Project administration*: Karen McBride‐Henry and Charissa Miller. *Supervision*: Karen McBride‐Henry. *Writing*: Karen McBride‐Henry, Charissa Miller and Tara N. Officer.

## Data Availability

Data sharing not applicable to this article as no datasets were generated or analysed during the current study.
